# Cross talk between cytokine and hyperthermia-induced pathways: identification of different subsets of NF-**κ**B-dependent genes regulated by TNFα and heat shock

**DOI:** 10.1007/s00438-015-1055-1

**Published:** 2015-05-06

**Authors:** Patryk Janus, Tomasz Stokowy, Roman Jaksik, Katarzyna Szoltysek, Luiza Handschuh, Jan Podkowinski, Wieslawa Widlak, Marek Kimmel, Piotr Widlak

**Affiliations:** Maria Sklodowska-Curie Memorial Cancer Center and Institute of Oncology, Gliwice Branch, Wybrzeze Armii Krajowej 15, Gliwice, Poland; Faculty of Automatic Control, Electronics and Computer Sciences, Silesian University of Technology, Akademicka 16, Gliwice, Poland; Institute of Bioorganic Chemistry, Polish Academy of Sciences, Noskowskiego 12/14, Poznan, Poland; Department of Hematology and Bone Marrow Transplantation, Poznan University of Medical Sciences, Szamarzewskiego 84, Poznan, Poland; Department of Statistics, Rice University, 6100 Main Street, Houston, TX USA; Institute of Automatic Control, Silesian University of Technology, Akademicka 16, Gliwice, Poland; Department of Clinical Science, University of Bergen, Postboks 7804, Bergen, Norway

**Keywords:** ChIP-seq, HSF1, Heat shock, Microarrays, NF-κB, Transcription factor binding

## Abstract

Heat shock inhibits NF-κB signaling, yet the knowledge about its influence on the regulation of NF-κB-dependent genes is limited. Using genomic approaches, i.e., expression microarrays and ChIP-Seq, we aimed to establish a global picture for heat shock-mediated impact on the expression of genes regulated by TNFα cytokine. We found that 193 genes changed expression in human U-2 osteosarcoma cells stimulated with cytokine (including 77 genes with the κB motif in the proximal promoters). A large overlap between sets of genes modulated by cytokine or by heat shock was revealed (86 genes were similarly affected by both stimuli). Binding sites for heat shock-induced HSF1 were detected in regulatory regions of 1/3 of these genes. Furthermore, pre-treatment with heat shock affected the expression of 2/3 of cytokine-modulated genes. In the largest subset of co-affected genes, heat shock suppressed the cytokine-mediated activation (antagonistic effect, 83 genes), which genes were associated with the canonical functions of NF-κB signaling. However, subsets of co-activated and co-repressed genes were also revealed. Importantly, pre-treatment with heat shock resulted in the suppression of NF-κB binding in the promoters of the cytokine-upregulated genes, either antagonized or co-activated by both stimuli. In conclusion, we confirmed that heat shock inhibited activation of genes involved in the classical cytokine-mediated functions of NF-κB. On the other hand, genes involved in transcription regulation were over-represented in the subset of genes upregulated by both stimuli. This suggests the replacement of NF-κB-mediated regulation by heat shock-mediated regulation in the latter subset of genes, which may contribute to the robust response of cells to both stress conditions.

**Electronic supplementary material:**

The online version of this article (doi:10.1007/s00438-015-1055-1) contains supplementary material, which is available to authorized users.

## Introduction

A subset of signal transduction pathways that respond to environmental stress cues is responsible for cell survival and adaptation. Regulation of these signaling pathways is critical for development of cancer and other human diseases and for responses to the treatments. Among the major pathways determining cellular responses to stress are those involving the NF-κB and HSF1 transcription factors. While the interactions within each of these pathways and their importance for cells have been extensively described, much less is known about the cross talk between them, which is the focus of the present work.

The NF-κB-dependent pathway mediates cell responses to different types of stimuli, yet its primary function is the coordination of inflammatory and immune responses such as expression of cytokines (Vallabhapurapu and Karin 2009). Other genes, whose transcription depends on NF-κB, include those encoding proteins involved in apoptosis (mainly anti-apoptotic proteins), activation of cell cycle progression, angiogenesis, and metastasis (e.g., adhesion molecules). In general, the κB-responsive element is found in the regulatory regions of several hundreds of such target genes. Upregulation of the NF-κB pathway is frequently observed in cancer cells, which contributes to their resistance to the anticancer treatment (Hayden and Ghosh 2012; Perkins 2012). NF-κB transcription factors are dimers formed by the members of the multigene NFκB/Rel family, which in humans includes five proteins. The RelA/p50 heterodimer is the major regulator in the so-called canonical NF-κB pathway, which is involved in inflammation and responses to cytokines (Tian and Brasier 2003). In the resting cells, NF-κB dimers are sequestered in the cytoplasm via binding with the inhibitory proteins IκB. Pro-inflammatory extracellular signals or cellular stress can induce activation of the IκB kinase (IKK) complex, which in turn phosphorylates the IκB protein causing its ubiquitination and degradation by the proteasome. This allows the translocation of RelA/p50 to the nucleus and its binding to κB regulatory elements in the promoters of the target genes. Expression of IκBα (the major inhibitor of NF-κB) is controlled by an NF-κB-responsive promoter, which generates the major internal circuit of autoregulation of NF-κB signaling (Perkins 2007). Activation of the canonical NF-κB pathway is rapid and transient. On the other hand, the non-canonical NF-κB pathway depends on the activation of the RelB/p52 heterodimer, which is much slower. The mature p52 subunit is generated from its precursor protein p100, whose processing is mediated by NIK (NF-κB inducing kinase) and IKKα after stimulation of cells; TNFα does not activate this pool of NF-κB (Sun 2012).

Heat shock response (HSR) arises under several types of cellular stress that lead to the denaturation of proteins, which in turn results in accumulation of heat shock proteins (HSPs). HSPs are the major molecular chaperones: during synthesis they assist in proper protein folding, while during proteotoxic stress they protect stress-labile proteins and contribute to the proteolysis of damaged species. Besides inducible HSPs, such as the most abundant HSPA1 (HSP70i), other members of the HSP superfamily are expressed also in the absence of stress. Constitutively expressed and inducible HSPs perform distinct physiological functions for cellular maintenance and adaptation to stress, respectively (Kampinga et al. 2009; Rupik et al. 2011). Heat shock transcription factor 1 (HSF1) is responsible for triggering stress-induced activation of the *HSP* genes (Anckar and Sistonen 2011). In addition to the activation of *HSP* genes, HSF1 is involved in the regulation of many other genes associated with multiple cellular processes including cell signaling, development, fertility, cell death, and metabolism (Page et al. 2006; Kus-Liśkiewicz et al. 2013). Increased expression of HSF1 and HSP proteins, which is observed in several tumors and cancer cell lines, may lead to cancer chemo- and radio-resistance. It also supports tumor growth and neoplastic transformation (Vydra et al. 2014). Under physiological conditions, HSF1 monomers exist in the cytoplasm in complexes with different HSPs. Upon proteotoxic stress, HSPs are released from such complexes and free HSF1 forms trimers that translocate to the nucleus. HSF1 trimers are further phosphorylated and then bind their target HSE-regulatory DNA sequences (Voellmy 2004).

The two major stress–response pathways, HSR and NF-κB, interfere with each other in a number of ways (Knowlton 2006; Salminen et al. 2008). Following heat shock, cells usually do not exhibit cytokine-induced degradation of IκB, nuclear translocation of NF-κB and the activation of the typically NF-κB-dependent genes (Feinstein et al. 1996; Wong et al. 1997a, b; Ayad et al. 1998). Several lines of evidence indicate that interactions of HSPs with IKK and/or NF-κB/IκB complexes may be primarily responsible for blocking of the NF-κB cascade (Park et al. 2003; Ran et al. 2004; Weiss et al. 2007; Zheng et al. 2008). A numerical model of cross talk between HSR and NF-κB signaling has been recently devised based on the importance of interactions of HSPA1, IKK, RelA, and IκBα (Sheppard et al. 2014). Moreover, interactions between HSPs and components of membrane receptor complexes upstream of IKK (like TRAF6 or TRAF2) have been observed (Chen et al. 2006; Dai et al. 2010). However, it has also been reported that activation of inducible HSPs by constitutively active HSF1 mutant in the absence of heat shock does not suppress activation of the NF-κB pathway by TNFα (Janus et al. 2011), and that HSPs can stabilize and facilitate renaturation of IKK after heat shock (Lee et al. 2005; Jiang et al. 2011). Hence, other mechanisms of interference between HSR and NF-κB should also be considered. For example, evidence for heat shock-induced aggregation and inactivation of IKK has been presented (Lee et al. 2004). It is also noteworthy that an alternative mechanism of NF-κB activation during the recovery from heat shock has been observed (nuclear translocation of NF-κB started after 3 h of recovery at “normal” temperature): in this case NF-κB was activated without prior IKK-dependent phosphorylation and degradation of IκB (“thermolability” of NF-κB/IκB complexes was proposed) (Kretz-Remy et al. 2001; Nivon et al. 2012). Understanding the interactions between the HSR and NF-κB pathways is further complicated by the fact that HSF1 can directly and/or indirectly influence transcription of specific NF-κB-dependent genes. Some of these genes are known to contain HSEs in their regulatory regions, e.g., binding of HSF1 to these elements is crucial for downregulation of endotoxin-activated *TNFA* (Singh et al. 2002) and upregulation of *NOS2* (Goldring et al. 2000). HSF1 binding can also modulate the chromatin structure of the *IL6* gene promoter, which facilitates the binding of other activators or repressors (Inouye et al. 2007). In addition, HSF1 can suppress the activity of some NF-κB-dependent genes (e.g., *IL6*) indirectly through the activation of ATF3, a negative transcriptional regulator of pro-inflammatory genes (Takii et al. 2010). Hence, one should expect pleiotropic effects of heat shock on regulation of NF-κB-dependent genes, which has not been addressed systemically at the level of complete gene expression pattern yet.

The aim of this study is to obtain a global picture of potential cross talk between heat shock and cytokine-modulated expression of NF-κB-dependent genes. Human U-2 osteosarcoma was selected as a model, because in this cell line both TNFα cytokine and heat shock revealed low toxicity and induced classical pathways of response. Moreover, the U-2 cells were used previously in several other studies that investigated regulation of the NF-κB pathway (Vertegaal et al. 2000; Bednarski et al. 2009; Janus et al. 2011). Cells were subjected to heat shock followed by stimulation with TNFα cytokine, and to both treatments separately. Our results revealed predictable inhibitory effects of heat shock on the expression of classical NF-κB-target genes, but also novel patterns of activation (or co-activation) and repression (or co-repression) for some gene subsets.

## Materials and methods

### Experimental model and treatments

Human osteosarcoma U-2 OS cells were purchased from ATCC, ref. no. HTB-96, and grown at 37 °C and 5 % CO_2_ in McCoy’s medium supplemented with 10 % heat-inactivated fetal bovine serum. All treatments were started 3–5 days after inoculation of cells. To induce the heat shock (HS) response, cells were incubated for 1 h in a water bath at 43 °C and then shifted to 37 °C for recovery. To induce the NF-κB pathway, cells were incubated for 30 min in a medium containing 10 ng/ml of TNFα cytokine (T0157; Sigma), and then the TNFα-containing medium was replaced with fresh TNFα-free medium. The viability of cells was analyzed 3 and 24 h after the end of heat shock or at the corresponding time after stimulation with TNFα. Cells were harvested, stained with propidium iodide, and analyzed on a FACS Canto cytometer (Becton–Dickinson). Then the percentage of viable and dead cells was calculated. All experiments were performed at least in triplicate. We found that all types of treatments (i.e., HS, TNFα, and their combination) were well tolerated, and slightly increased toxicity was observed only when heat shock was followed by stimulation with TNFα (see supplementary Fig. S1).

### Gene expression analysis

Cells, either without heat shock pre-conditioning or directly after heat treatment, were stimulated with the TNFα cytokine (for 30 min) and harvested 90 min after cytokine removal (which corresponds to the “early phase” of the NF-κB response; Tian et al. 2005). Alternatively, cells were harvested 2 h after the end of HS alone. RNA was isolated from cells using Total RNA Isolation kit (A&A Biotechnology) and purified by DNase I digestion. Two hundred ng of RNA was processed using GeneChip 3′IVT Express Kit (Affymetrix) according to the manufacturer’s protocol and then analyzed using GeneChip Human Genome U133a 2.0 Plus Array (Affymetrix). RNA from two biological experiments was combined, and then three technical replicas of microarray analyses were performed. Microarray data quality was evaluated using the Bioconductor via the Simpleaffy package (Wilson and Miller 2005). Statistical data pre-processing was conducted using GC-RMA approach (Wu et al. 2004), based on Brainarray, EntrezGene specific CDF file (ver. 15) (Dai et al. 2005). Differentially expressed genes were identified using Limma software package (Smyth 2004) with correction for multiple testing using false discovery rate (FDR) estimation (Storey and Tibshirani 2003); at FDR = 0.05. Furthermore, only genes whose expression levels changed by more than 20 % of control levels were taken into consideration (corresponding to signal ratio >1.2 or <0.8).

### Chromatin immunoprecipitation

To induce chromatin binding of HSF1 or NF-κB, cells were treated at 43 °C for 10–20 min or incubated with TNFα for 30 min, respectively. Cells were fixed with formaldehyde added directly to the growth medium (1 % final concentration), scraped mechanically, and then incubated for 10 min at 37 °C with dish rotation. Formaldehyde fixation was stopped by adding glycine (125 mM final concentration) and incubation for 5 min at 37 °C with rotating. Fixed cells were collected by centrifugation (4 min at 1700*g*), washed twice with ice cold PBS, and then incubated with lysis buffer (consisting of 50 mM Tris pH 7.4, 250 mM NaCl, 5 mM EDTA, 50 mM NaF, 1 mM Na_3_VO_4_, 1 % NP40, 0.02 % NaN_3_, and Complete™ Roche protease inhibitor cocktail) for 30 min on ice (with vortexing every 10 min). Nuclei were collected by centrifugation (10 min at 400*g*), suspended in a fresh lysis buffer, and then sonicated for chromatin fragmentation using Ultrasonic Processor CP-130 (ten times, output power equal to 5 W)—DNA fragments of 200–800 bp of length were obtained. Chromatin fragments were subjected to ChIP reaction using Dynabeads^®^ Protein A Immunoprecipitation Kit (Novex) without antibody (mock probe) or with anti-HSF1 (cat. no. ADI-SPA-901, Enzo Life Sciences), or anti-RelA(p65) (ab7970; Abcam) antibodies; 1 µg Ab for every 10 µg of chromatin was used. Protein–DNA cross links were reversed by 6 h incubation at 60 °C, then DNA was purified by proteinase K treatment, phenol/chloroform extraction, and ethanol precipitation. To validate HSF1-ChIP reaction, three genes known for HSF1 binding (*HSPA1A, HSPH1, TNF*) were analyzed using QRT-PCR. To analyze binding of RelA(p65) NF-κB subunit, QRT-PCR reaction was also used; κB motifs in individual genes were identified using rVista database (http://rvista.dcode.org). For information about the sequence of all PCR primers and amplified regions, see Supplementary file 1.

### ChIP-Seq analysis of HSF1 binding

To analyze the global pattern of HSF1 binding, immunoprecipitated DNA fragments (and input DNA) were sequenced using the TruSeq System and Genome Analyzer II (Illumina). DNA libraries were obtained using ChIP-Seq DNA Sample Prep Kit I (Illumina) and gel-purified using QIAquick Gel Extraction Kit (Qiagen). Sequences were read using the TruSeq SR Cluster Kit v2 cBot-GA and the TruSeq SBS Kit v5 GA (Illumina). Raw sequencing reads were analyzed according to standards of ChiP-Seq data analysis as follows. Quality control of reads was performed with FastQC software (http://www.bioinformatics.babraham.ac.uk/projects/fastqc) and low-quality sequences (average phred <30) were filtered out. The remaining reads were aligned to the reference human genome sequence (hg19) using the Bowtie2.0.4 program (Langmead and Salzberg 2012). Peak detection was carried out with the MACS program (Feng et al. 2012), whereas the outcome was annotated with the Homer package (Heinz et al. 2010). Peak intersections and their genomic coordinates were found using Bedtools software (Quinlan and Hall 2010). The input DNA was used as a reference because no sequences were obtained using mock-IP probe. The significance of differences between control untreated cells and cells subjected to HS (for 10 and 20 min) was estimated using MACS software; FDR = 0.05 level was selected as the significance threshold.

### Transcription factor-binding motif prediction and gene ontology analyses

For all 18,904 genes (for which transcript signals were detected on microarray’s chip), bioinformatics prediction of potential κB and HSE motifs was performed using *g:Profiler* server (gene ontology tool) (Reimand et al. 2007), which for this purpose uses binding motif patterns from the TRANSFAC database (Matys et al. 2003). Gene lists were annotated with GO terms using NucleoAnnot software (http://www.cellab.polsl.pl); the significance of the term overrepresentation was evaluated using the hypergeometric test (*p* < 0.05 was used as the significance threshold level).

## Results

### Transcription of the majority of TNF-regulated genes is also affected by heat shock

**Fig. 1:**
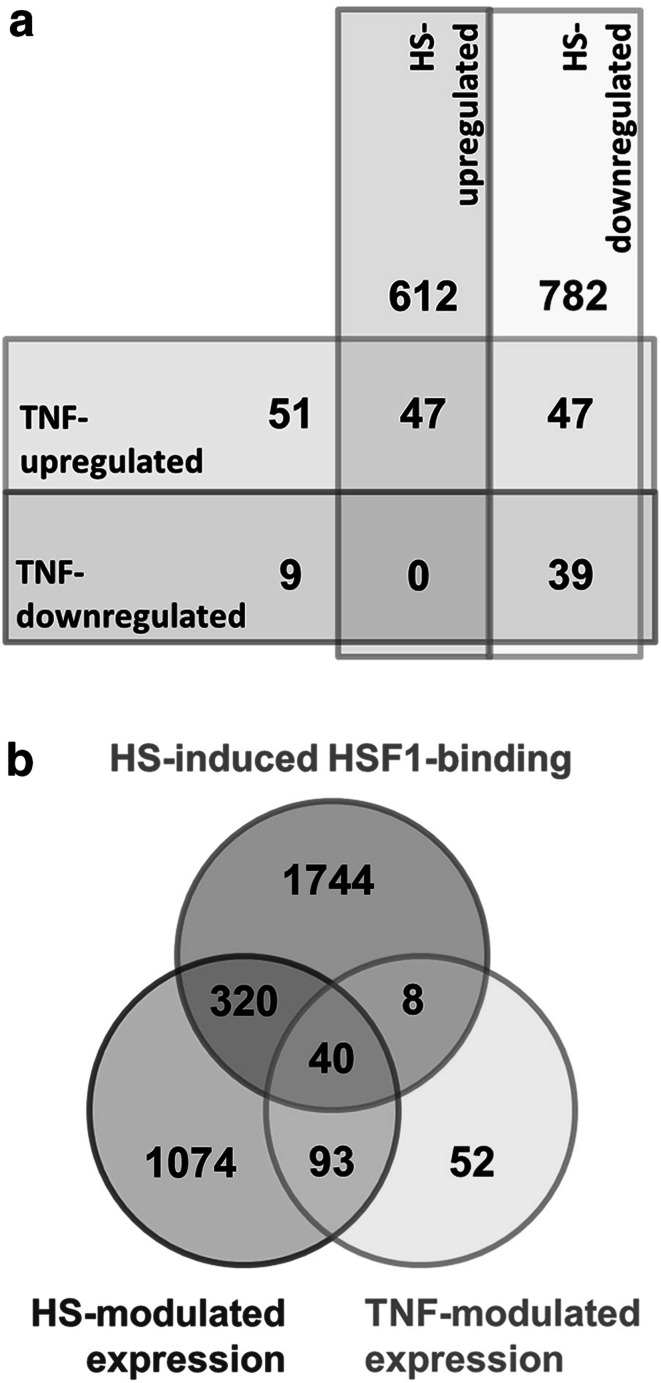
Numbers of genes, with expression modulated by TNFα cytokine or heat shock in U-2 OS cells. **a** Genes upregulated and downregulated by either TNFα or heat shock. **b** Genes modulated by TNFα and/or heat shock and containing actual binding sites for HS-induced HSF1 in their regulatory regions

To identify the novel sets of NF-κB-dependent genes that may be co-regulated by heat shock, we characterized the global gene expression patterns in U-2 OS cells. Expression of typical NF-κB-regulated genes was studied after stimulation with TNFα cytokine, which activates the classical (canonical) NF-κB response in these cells. The classical heat shock (HS) response was activated upon incubation of cells at elevated temperature as described in “Materials and methods”. The expression levels of 18,904 genes whose transcript signals were recorded are presented in Table S1 in Supplementary file 2; changes (treatments versus control) were considered significant if signal ratios were >1.2 or <0.8 and FDR <0.05. We observed that 193 genes met these criteria and changed their expression upon stimulation with TNFα, while 1527 genes were affected in response to HS. Next, we identified a set of genes, whose expression could be modulated by either TNFα or HS used as independent stimuli. We found that the majority of genes modulated by TNFα (133 out of 193) could be also affected by HS (Fig. 1a). More specifically, 47 genes were upregulated, while 39 genes were downregulated by both TNFα and HS. For all genes whose transcripts were detected, we performed bioinformatics prediction of potential binding sites for NF-κB and HSF1 transcription factors (hereafter termed κB and HSE motifs, respectively); hypothetical binding motifs were searched in regions spanning from −1000 bp to the transcription start sites, TSS, i.e., in the “proximal promoter regions”. The κB motifs were found in proximal promoters of 4542 genes (i.e., ca. 24 % of all analyzed genes), including 77 genes, whose transcription was modulated by TNFα (i.e., ca. 40 % of TNF-modulated genes; this group putatively represents the set of genes regulated by TNFα via the NF-κB pathway), and also 321 HS-modulated genes. The HSE motifs were found in the proximal promoters of 1188 genes (i.e., ca. 6 % of all analyzed genes), including 118 HS-modulated genes, and also 17 TNF-modulated genes (see Table S2 in Supplementary file 2). In the group of genes modulated by both TNFα and HS, 54 genes contained the κB motif and 11 genes contained the HSE motifs. Hence, a large overlap between sets of genes stimulated by cytokine or heat shock was revealed.

### HSF1 binding sites can be detected in the regulatory regions of several TNF-modulated genes

In the next step we aimed to identify binding sites of heat shock-induced HSF1 in regulatory regions of TNF/NF-κB-dependent genes. To validate the experimental model, the binding of HSF1 to HSE motifs in promoters of classical heat shock-induced *HSP* genes (*HSPA1A* and *HSPH1)* and a putative HSF1-target *TNF* gene was confirmed using a ChIP-PCR approach in heat-shocked cells (supplementary Fig. S2). The global pattern of HSF1 binding was analyzed after 10 and 20 min of HS using the ChIP-Seq approach (complete dataset is available at the GEO database, accession no. GSE60984). The analysis revealed about 25000 HSF1 binding sites in the chromatin of control untreated U-2 OS cells (i.e., cells cultured at 37 °C), which nearly doubled in cells subjected to heat shock. Functional binding sites can be found in several thousand base pairs from TSS, and this distance may determine the mode of the transcription factor’s activity (Cooper et al. 2006). Hence, in our analyses we defined the regulatory region as a region spanning from −7500 to +2500 base pairs from the TSS (similar to a promoter array approach), which apparently contains distal promoters and other potential regulatory elements. About 6000 HSF1 binding sites were found in such potential regulatory regions when control untreated cells were analyzed, which nearly doubled in cells subjected to HS (supplementary Fig. S3a). In general, heat shock resulted in increased binding of HSF1 in regulatory regions of 2104 genes (i.e., ca. 11 % of all analyzed genes; see supplementary Fig. S3b). We found HS-induced binding of HSF1 in regulatory regions of about 24 % of HS-modulated genes, including 240 upregulated and 120 downregulated genes. It is noteworthy that binding of HS-induced HSF1 was detected in regulatory regions of 48 TNF-modulated genes (i.e., ca. 25 % of such genes), including 28 genes containing the κB motif in their proximal promoters (examples of HS-induced binding of HSF1 in regulatory regions of HS-responsive or TNF-modulated genes are shown in supplementary Fig. S4). Furthermore, the HSF1 binding sites were detected in regulatory regions of about 30 % of genes that could be affected by both TNFα and HS (Fig. 1b, also see Table S2 in Supplementary file 2). Hence, the binding of HSF1 in regulatory regions of TNF/NF-κB-modulated genes was more frequent than the genome average.

### Heat shock pre-conditioning affects expression of several TNF-modulated genes

**Fig. 2:**
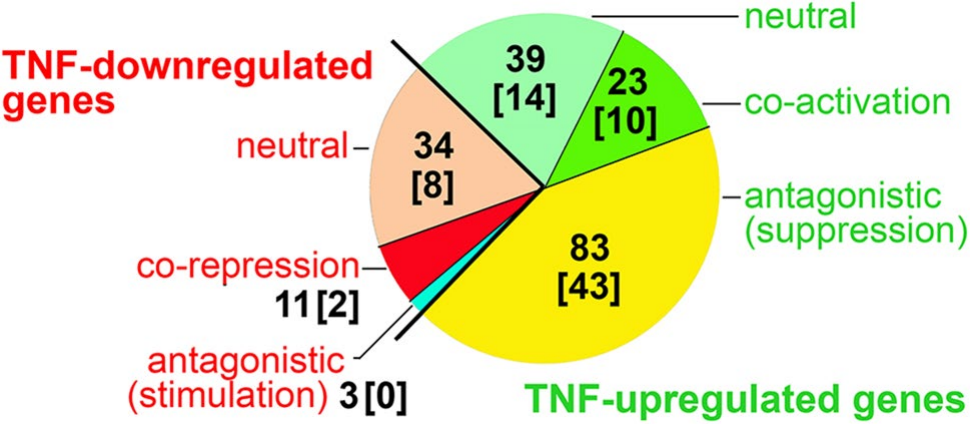
Numbers of cytokine-modulated genes, with expression affected by pre-treatment with the heat shock. The cytokine-upregulated/downregulated genes and different effects mediated by the heat shock are shown; the numbers of genes containing the κB motifs in their promoters are placed in *square parentheses*

The data presented above collectively show that expression of a large set of TNF-modulated genes was also affected by heat shock, which indicated the possibility of their co-regulation by both treatments. Furthermore, we tested whether 1 h exposure to HS prior to stimulation with TNFα (i.e., HS pre-treatment) affects expression of cytokine-modulated genes. In general, we observed that after such combined treatment, the expression of 1156 genes was different when compared with the cytokine treatment alone, yet the vast majority of such differences (for >80 % of genes) could be attributed to the modulatory effect of heat shock itself (see Table S1 in Supplementary file 2). Next, we focused on a set of 193 TNF-modulated genes (Fig. 2). We found that expression of ca. 62 % of such genes was affected by the heat shock pre-treatment (i.e., expression after TNF treatment alone was different then expression after TNF treatment preceded by HS). Four subsets of such “co-affected” genes could be distinguished, whose expression: (1) upregulated by TNFα alone was further increased after HS pre-treatment; (2) downregulated by TNFα alone was further decreased after HS pre-treatment; (3) upregulated by TNFα alone was suppressed after HS pre-treatment; (4) downregulated by TNFα alone was enhanced after HS pre-treatment (ca. 12, 6, 43, and 1.5 % of TNF-modulated genes, respectively). The former two modes represent putative co-activation and co-repression by both stimuli, while the latter two modes could be called antagonistic (opposite). It is noteworthy that the majority of genes co-affected by heat shock and cytokine (83 out of 120) showed the antagonistic effects of pre-exposure to HS that suppressed or inhibited subsequent activation by TNFα. More than half of such genes (47 genes) were downregulated by HS alone, yet others were either not affected (34 genes) or even activated (2 genes) by heat shock itself. Furthermore, genes co-activated and co-repressed by the combined treatment were upregulated and downregulated, respectively, by both treatments alone. Specifically, all 23 co-activated genes belong to the subset of 47 upregulated genes, while all 11 co-repressed genes belong to the subset of 39 downregulated genes (see Table S2 in Supplementary file 2). Hence, in the largest subset of TNF-modulated genes, pre-treatment with HS suppressed the capability for cytokine-mediated activation, yet subsets of putatively co-activated and co-repressed genes were also revealed. Moreover, we analyzed the influence of the heat shock extension on the expression of TNF-upregulated genes and found that 10 or 20 min HS pre-treatment markedly reduced the activation of TNF-responsive genes, yet full inhibition of target genes required 1 h HS pre-treatment (supplementary Fig S5). We also searched for a hypothetical influence of cytokine on the expression of the HS-modulated genes. We found that very few HS-modulated genes (17 upregulated and 25 downregulated) showed different expression when combined treatment (i.e., 1 h heat shock followed by cytokine stimulation) and HS alone were compared, including 15 genes affected by TNFα alone (see Table S1 in Supplementary file 2). Thus, in a marked contrast to the very strong effect of heat shock on expression of TNF-modulated genes, only minimal effect of TNFα post-treatment on general expression of HS-modulated genes was observed.

### TNFα cytokine and heat shock co-affect genes involved in immune responses, stress responses, and regulation of transcription

To identify biological processes and molecular functions of genes, whose expression was modulated by TNFα, we annotated them at the Gene_Ontology (GO) knowledge base. Among biological and molecular functions statistically over-represented in this set of genes, we found those related to the regulation of transcription, inflammation, immune responses, regulation of cell proliferation, regulation of apoptosis, response to stress, and other types of stimuli (e.g., drugs or hypoxia). As expected, similar GO terms were enriched in different subsets of TNF-modulated genes. Nevertheless, we looked for GO terms associated with three smaller subsets of TNF-regulated genes: 83 genes activated by cytokine and suppressed (antagonized) by the heat shock pre-treatment, as well as 47 genes upregulated by both cytokine and heat shock, and 39 genes downregulated by both treatments used separately. Statistical probability of over-representation was compared between these smaller subsets of genes and the overall set of TNF-modulated genes (193 genes). We found that several GO terms showed a significantly higher probability of over-representation in the subset of heat shock-antagonized genes compared to the overall set of TNF-modulated genes, which included terms involved in inflammation and bacteria response, chemokine and cytokine response, membrane-mediated signaling, and regulation of apoptosis (Table S3 in Supplementary file 2). Moreover, this subset contained all key genes involved in auto-regulation of the NFκB circuit (namely *NFKB1, NFKBIA, NFKBIE, REL, RELB, TNF, TNFAIP3*). On the other hand, a large group of GO terms (7 out of 20) over-represented in a subset of genes upregulated by both cytokine and heat shock was associated with regulation of transcription. Additionally, GO terms over-represented in this subset of genes included terms involved in response to different types of stress and hormone stimuli (Table S4 in Supplementary file 2). On the contrary, no specific group of GO terms was over-represented in the subset of genes downregulated by both treatments (Table S5 in Supplementary file 2). These results suggest the apparent selectivity of TNF/NF-κB-regulated processes/functions that are modulated differently in a heat shock-dependent manner.

To address another potential aspect of cross talk between the NF-κB pathway and heat shock, we analyzed 3665 genes that contained κB motif, but neither HSE motif nor HS-induced HSF1 binding site in their regulatory regions. Among them there were 100 genes upregulated by heat shock (including 5 genes upregulated by TNFα) and 193 genes downregulated by heat shock (including 9 genes downregulated and 20 genes upregulated by TNFα) (see Table S1 in Supplementary file 2). Hence, expression of ca. 8 % of such genes was affected by heat shock (most of them were downregulated). This observation further confirmed that genes potentially regulated by NF-κB are likely inhibited by heat shock even in the absence of HSF1 binding. Analysis of GO terms associated with a subset of HS-downregulated genes containing κB motif in their promoters was performed in comparison to a complete set of genes containing κB motif, but neither HSE motif nor HSF1 binding site. Interestingly, among 41 GO terms over-represented in the subset of HS-downregulated genes, there were 16 terms related to regulation of gene expression and transcription (Table S6 in Supplementary file 2). On the other hand, expression of only ca. 1 % of genes that contained either the HSE motif or HSF1 binding site, but not κB motif in their regulatory regions was affected by TNFα cytokine (see Table S1 in Supplementary file 2), which indicated the marginal influence of TNFα cytokine on the expression of HSF1-regulated genes.

### NF-κB and HSF1 binding sites are present in the regulatory regions of several genes affected by TNFα and heat shock

Among 145 genes upregulated by TNFα, there were 27 genes containing the κB motif in proximal promoters and HSF1 binding sites in their regulatory regions (genes listed in Table 1), which indicated possible involvement of both NF-κB and HSF1 transcription factors in their regulation and made this subset of genes particularly interesting. Among them, 9 genes were downregulated and 13 upregulated by heat shock alone. When genes co-affected by heat shock and cytokine were analyzed, seven genes were co-activated (namely, *EGR1, EGR2, FOSB, MSX1, PPP1R15A, RRAD,* and *ZFP36*), which were also upregulated by both TNFα and heat shock when cells were exposed to these treatments separately. Furthermore, 14 genes were antagonized by both treatments (namely, *BTG2*, *CCL2*, *CCL20*, *CD83*, *CYR61*, *DUSP2*, *EGR4*, *ETS1*, *IFNGR2*, *IL8*, *INHBA*, *KCTD11*, *SLC12A7*, and *TNF*). However, this antagonistic effect (i.e., inhibition by the heat shock pre-treatment) was not predetermined by the type of effect of heat shock alone. It is noteworthy that among the above-mentioned genes potentially co-regulated by NF-κB and HSF1, there are a few NF-κB-dependent genes whose binding of HSF1 and/or regulation by heat shock had been previously reported. HSE-like sequences and HSF1 binding have been documented in the 5′UTR of the *TNF* gene, whose LPS induction was reduced at febrile temperature (Singh et al. 2000, 2002). HSF1 binding has been observed in the promoter of the *IL8* gene, which has been linked with the co-activation of this gene in epithelial cells stimulated with TNFα and then exposed to heat shock (Singh et al. 2008). Involvement of HSF1 in the regulation of gene expression has been also suggested for *PPP1R15A* (Hensen et al. 2013) and *EGR1* (Trinklein et al. 2004). Additionally, heat shock-induced binding of HSF1 in promoter regions of the *CCL20* and *EGR4* genes has been reported in a genome-wide ChIP-microarray study (Page et al. 2006).

**Table 1:** Subset of TNFα-upregulated genes containing the κB motifs and the HSF1 binding sites in the regulatory regions

Gene symbol	Modulation by HS alone	HS_TNF/TNF signal ratio	Combined effect of TNF and HS
*CCL2*	▼	0.03	O
*CCL20*	▼	0.01	O
*CYR61*	▼	0.34	O
*DUSP2*	▼	0.24	O
*ETS1*	▼	0.52	O
*IFNGR2*	▼	0.66	O
*IL8*	▼	0.05	O
*INHBA*	▼	0.28	O
*KCTD11*	▼	0.40	O
*EGR4*	▲	0.64	O
*SLC12A7*	▲	0.76	O
*BTG2*		0.46	O
*CD83*		0.35	O
*TNF*		0.77	O
*EGR1*	▲	1.66	A
*EGR2*	▲	1.36	A
*FOSB*	▲	2.61	A
*MSX1*	▲	4.14	A
*PPP1R15A*	▲	2.11	A
*RRAD*	▲	19.23	A
*ZFP36*	▲	1.80	A
*ATF3*	▲	1.00	
*EPB41L2*	▲	0.97	
*GADD45B*	▲	0.87	
*NR4A1*	▲	0.85	
*AZIN1*		0.93	
*BDKRB1*		0.63	

Down and up arrowheads represent transcriptional downregulation and upregulation, respectively, by heat shock (HS). Combined effect of HS prior to TNF is marked with O (opposite/antagonistic) and A (co-activation)

### Heat shock pre-treatment suppresses binding of NF-κB in the promoters of the TNFα-stimulated genes

**Fig. 3:**
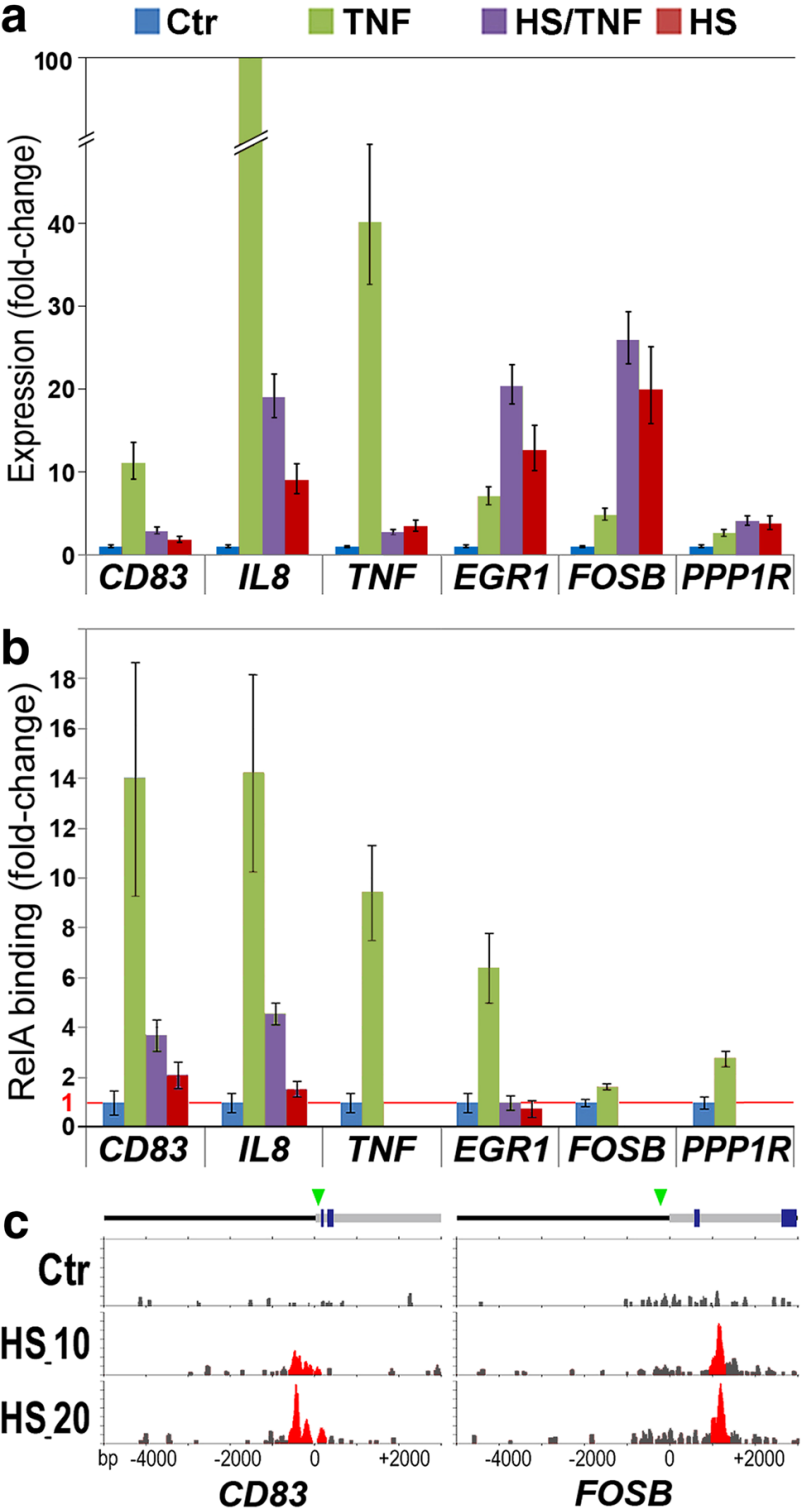
The influence of the heat shock on the expression and promoter binding of NF-κB in selected cytokine-upregulated genes. **a** Relative gene expression assessed by QRT-PCR in cells treated with TNFα cytokine (TNF) or heat shock (HS), or conditioned with heat shock prior to TNF stimulation (HS/TNF), is presented as a fold change against untreated controls (Ctr). The mean values ± SD are shown. **b** Relative binding of RelA(p65) assessed by ChIP-QPCR is presented as a fold change against untreated controls (Ctr); the mean values ± SD are shown. **c** Examples of heat shock-induced HSF1 binding sites in regulatory regions of *CD83* and *FOSB* genes; the HSF1 peaks detected by ChIP-Seq in control and heat-shocked cells (HS_10 and HS_20), exons (represented by *blue bars*), and NF-κB binding sites (represented by *green arrowheads*) are shown (color figure online)

Finally, we assessed the possible influence of heat shock pre-conditioning on the binding of NF-κB in promoters of target genes. Six NF-κB-dependent genes were selected for a gene-specific ChIP analysis: three genes represented a subset of heat shock/cytokine antagonized genes (*CD83*, *IL8*, and *TNF*), while the other three genes represented a subset of heat shock/cytokine co-activated genes (*EGR1*, *FOSB*, and *PPP1R15A*) (Fig. 3a). Binding of NF-κB species containing the RelA(p65) subunit, which is critical for the canonical NF-κB pathway, was analyzed in the known κB motifs present in the regulatory regions of these genes. For all six analyzed genes, stimulation with TNFα significantly increased binding of RelA, which correlated with the upregulation of their transcription, while pre-treatment with heat shock markedly reduced or fully inhibited such cytokine-stimulated binding; also heat shock itself did not induce such binding (Fig. 3b). Notably, the heat shock-mediated suppression of RelA binding was observed also in co-activated genes upregulated by either cytokine, heat shock, or the combined treatment. Importantly, the regulatory regions of all these genes contained binding sites for heat shock-activated HSF1 (Fig. 3c; supplementary Fig. S4). Hence, this observation suggested that heat shock/HSF1-mediated signaling could hypothetically replace NF-κB-mediated signaling in the case of heat shock/cytokine co-activated genes.

## Discussion

There are convincing reports of cells exposed to heat shock usually not exhibiting degradation of IκB and nuclear translocation of NF-κB after subsequent stimulation with cytokines (Feinstein et al. 1996; Wong et al. 1997a, b; Ayad et al. 1998). Several hypothetical mechanisms of heat shock-related inhibition of NF-κB signaling have been proposed, including: (1) inhibition of the IKK-mediated activation of NF-κB by HSPs, (2) inhibition of the cytokine receptors mediated by HSPs, and/or (3) thermal aggregation/inactivation of the components of the NF-κB pathway (Park et al. 2003; Lee et al. 2004; Ran et al. 2004; Chen et al. 2006; Weiss et al. 2007; Zheng et al. 2008; Dai et al. 2010; Sheppard et al. 2014). Another important question related to the cross talk between the HSR and NF-κB pathways is a role for heat shock-activated HSF1 itself. However, understanding the role of HSF1 is complicated by the fact that in addition to its canonical function as the primary transcription factor regulating HSR, this protein has several other cellular functions, including modulation of glucose metabolism, cell cycle progression, cell death, and drug efflux (Vydra et al. 2014), and beside its classical targets (i.e., *HSP* genes) HSF1 can also regulate (directly and/or indirectly) transcription of numerous other genes (Page et al. 2006; Kus-Liśkiewicz et al. 2013). In fact, certain NF-κB-dependent genes have been reported as targets for HSF1, some of which contain functional HSE sequences in their regulatory regions (Goldring et al. 2000; Singh et al. 2002; Inouye et al. 2007), which was confirmed and extended in this work. Studies on the influence of heat shock to modulate the NF-κB response usually have addressed limited numbers of classical NF-κB-dependent genes. Hence, a systemic knowledge of the influence of heat shock and heat shock-activated HSF1 on global expression patterns of NF-κB-dependent genes, which is the focus of our current work, has been missing so far.

In the present study, we found a substantial overlap between the set of genes potentially regulated by heat shock/HSF1 and the set of genes potentially regulated by TNFα/NF-κB. There were two subsets of genes similarly affected by TNFα and heat shock: 47 upregulated and 39 downregulated. Furthermore, the influence of heat shock on the expression of TNFα/NF-κB-dependent genes has been analyzed directly in cells conditioned with heat shock prior to stimulation with the cytokine, and a few subsets of TNF-upregulated genes could be distinguished based on the effects of the heat shock pre-treatment. The largest subset (83 genes, including 43 genes with the κB motif) consisted of genes, whose cytokine-dependent activation was suppressed after heat shock (the combined effect of both types of stimuli was called antagonistic). This observation could be expected assuming general suppression of NF-κB activation in cells subjected to heat shock, which was also observed in U-2 OS cells (Janus et al. 2011). Hence, the simplest mechanistic model for explaining the antagonistic effect of heat shock and cytokine stimulation might be blocking of the classical mechanism of NF-κB activation in cells pre-exposed to heat shock. In fact, we showed here examples of the heat shock-mediated inhibition of NF-κB binding in promoters of such genes. On the other hand, the group of genes “antagonized” by heat shock represented only a half of the genes upregulated by the cytokine. The expression of other TNF-upregulated genes was either unaffected or even further stimulated by the heat shock pre-treatment (when compared to cytokine activation alone). It is noteworthy that all genes co-activated by heat shock and TNFα were also upregulated in cells subjected to heat shock alone. Furthermore, their stimulation by heat shock alone was generally stronger than stimulation by cytokine alone and similar to the effects of the combined treatment (similar effects were also noted when a subset of co-repressed genes was analyzed). Additionally, we showed here examples of the heat shock-mediated inhibition of NF-κB binding and induction of HSF1 binding in promoters of such co-activated genes. Hence, these observed effects could be explained by a simple replacement of the NF-κB-mediated mechanism by the heat shock-mediated mechanisms of gene regulation. Moreover, expression of several TNF-upregulated genes remained unchanged in spite of the heat shock pre-treatment, which could suggest the existence of heat shock-resistant alternative pathways of NF-κB signaling. In conclusion, our results collectively demonstrate that the heat shock-mediated suppression of cytokine-modulated pathways is not a widespread phenomenon and only pertains to specific subsets of NF-κB-regulated genes.

Finally, we inspected the functional roles of the genes co-affected by the heat shock/HSF1 and the cytokine/NF-κB treatments. Among functions associated with genes antagonized by heat shock (i.e., suppressed after the heat shock pre-treatment) were those representing canonical processes mediated by NF-κB signaling including auto-regulation circuits and humoral immune responses as well as chemokine and cytokine responses (Tian and Brasier 2003; Vallabhapurapu and Karin 2009). On the other hand, among functions associated with the genes co-activated by cytokine and heat shock were those related to responses to different types of stress and hormone stimuli. Furthermore, the GO terms associated with regulation of transcription were significantly over-represented in a subset of genes upregulated by both cytokine and heat shock (which included a smaller subset of co-activated genes). There were 13 known or putative transcription factors in this group, namely *ATF3, EGR1*, *EGR2,EGR4, FOSB*, *MSX1*, *NR4A1*, *ZFP36, CSRNP1, FOS*, *JUN, SIX4,* and *TBX3* (the first 8 contained both NF-κB and HSF1 binding sites in their promoters), some of them with obvious stress-related functions (Hess et al. 2004). Moreover, both the cytokine and heat shock downregulated nine other factors putatively involved in transcription, namely *ELF1, ERF, HMX2, NACC2, TSHZ3, WWTR1, ZNF133, ZNF627,* and *ZNF827*. Assuming “stability” of their expression after stimulation with either TNF, heat shock, or both, one should consider the importance of these transcription factor-encoding genes for mounting a robust response to different stress conditions.

Resistance of cancer cells to anticancer treatment is frequently associated with upregulation of the NF-κB pathway (Hayden and Ghosh 2012; Perkins 2012). On the other hand, elevated temperature can enhance the sensitivity of cells to radiation and anticancer drugs in the mechanism not directly related to expression of HSP, and hyperthermia could be applied as an adjuvant treatment together with radiotherapy and/or chemotherapy (Hildebrandt et al. 2002; Kampinga 2006). Hence, expanding the knowledge of cross talk between NF-κB pathways which can enhance resistance to treatment, and heat shock-induced mechanisms which can sensitize cells to treatment, could have relevance to optimization of anticancer strategies.

In conclusion, we found a substantial overlap of genes potentially regulated by the TNFα cytokine and heat shock, and subsets of genes upregulated or downregulated by both types of stimuli have been identified. Moreover, heat shock pre-conditioning inhibits cytokine-mediated activation of a subset of genes, which in most cases are associated with canonical functions of NF-κB signaling. However, the heat shock-induced suppression is not the only possible mechanism of cross talk between cytokine/NF-κB-mediated and heat shock-mediated pathways. Indeed, we have uncovered novel subsets of genes that exhibit similar cytokine-dependent and heat shock-dependent transcriptional regulation. These genes, including a large group of genes encoding for transcription factors, hypothetically contribute to the robustness of cells to cope with multiple pathways of stress.

## Electronic supplementary material

Supplementary material 1 Toxicity of heat shock and/or stimulation with TNFα cytokine to U-2 OS cells. Cells were collected 3 and 24 h after the end of HS (1 h at 43 °C), or at corresponding time points in untreated controls or cells stimulated with cytokine. Relative numbers of living and dead cells were assessed by flow cytometry after staining with propidium iodide; shown are the mean values ± SD based on three experiments (JPEG 154 kb)

Supplementary material 2 Binding of HSF1 in promoters of *HSPA1A*, *HSPH1*, and *TNF* genes. Relative binding of HSF1 (assessed by ChIP-QPCR) is shown as a fold-change against untreated control (Ctr); cells were analyzed after 10 and 20 min of heat shock—HS(10′) and HS(20′), respectively. Shown are the mean values ± SD based on three experiments (JPEG 168 kb)

Supplementary material 3 Numbers of functional HSF1 binding sites in chromatin of U-2 OS cells. **a** Total number of HSF1 binding sites in untreated control cells (Ctr) and cells exposed to heat shock for 10 or 20 min––HS_10′ and HS_20′, respectively; depicted are binding sites present in potential regulatory regions (from −2500 to +500 bp from TSS) and other genomic sites. **b** Heat shock-induced HSF1 binding in regulatory regions after either 10 or 20 min of heat shock; depicted are numbers of genes with binding sites where HSF1 binding was significantly increased compared to untreated control (FDR <0.05) (JPEG 240 kb)

Supplementary material 4 Heat shock-induced HSF1 binding sites in regulatory regions of HS-responsive genes *HSPA1A* and *HSPH1*, and TNF-responsive genes *IL8*, *TNF*, *EGR1* and *PPP1R15A*. Shown are HSF1 peaks detected by ChIP-Seq in control and heat-shocked cells (HS_10 and HS_20), exons (represented by thick blue bars) and NF-κB binding sites (represented by green arrowheads); statistically significant HS-induced HSF1 binding sites are marked in red. (JPEG 464 kb)

Supplementary material 5 The influence of heat shock extension on expression TNF-upregulated genes. Cells were stimulated with TNFα alone (TNF), heat-shocked at 43 °C for 10, 20 or 60 min, and then stimulated with TNFα (HS + TNF), or treated with HS alone for 10, 20 or 60 min. Expression of *IL8* and *TNF* genes was assessed by QRT-PCR and presented as a fold-change against untreated controls (Ctr); shown are the mean values ± SD (JPEG 242 kb)

Supplementary material 6 (DOC 47 Kb)

Supplementary material 7 (XLS 5702 kb)

## References

[CR1] Anckar J, Sistonen L (2011). Regulation of HSF1 function in the heat stress response: implications in aging and disease. Annu Rev Biochem.

[CR2] Ayad O, Stark JM, Fiedler MM, Menendez IY, Ryan MA, Wong HR (1998). The heat shock response inhibits RANTES gene expression in cultured human lung epithelium. J Immunol Baltim Md 1950.

[CR3] Bednarski BK, Baldwin AS, Kim HJ (2009). Addressing reported pro-apoptotic functions of NF-kappaB: targeted inhibition of canonical NF-kappaB enhances the apoptotic effects of doxorubicin. PLoS One.

[CR4] Chen H, Wu Y, Zhang Y, Jin L, Luo L, Xue B, Lu C, Zhang X, Yin Z (2006). Hsp70 inhibits lipopolysaccharide-induced NF-kappaB activation by interacting with TRAF6 and inhibiting its ubiquitination. FEBS Lett.

[CR5] Cooper SJ, Trinklein ND, Anton ED, Nguyen L, Myers RM (2006). Comprehensive analysis of transcriptional promoter structure and function in 1% of the human genome. Genome Res.

[CR6] Dai M, Wang P, Boyd AD, Kostov G, Athey B, Jones EG, Bunney WE, Myers RM, Speed TP, Akil H, Watson SJ, Meng F (2005). Evolving gene/transcript definitions significantly alter the interpretation of GeneChip data. Nucleic Acids Res.

[CR7] Dai S, Jiang L, Wang G, Zhou X, Wei X, Cheng H, Wu Z, Wei D (2010). HSP70 interacts with TRAF2 and differentially regulates TNFalpha signalling in human colon cancer cells. J Cell Mol Med.

[CR8] Feinstein DL, Galea E, Aquino DA, Li GC, Xu H, Reis DJ (1996). Heat shock protein 70 suppresses astroglial-inducible nitric-oxide synthase expression by decreasing NFkappaB activation. J Biol Chem.

[CR9] Feng J, Liu T, Qin B, Zhang Y, Liu XS (2012). Identifying ChIP-seq enrichment using MACS. Nat Protoc.

[CR10] Goldring CE, Reveneau S, Chantome A, Pance A, Fleury C, Hume DA, Sester D, Mignotte B, Jeannin JF (2000). Heat shock enhances transcriptional activation of the murine-inducible nitric oxide synthase gene. FASEB J Off Publ Fed Am Soc Exp Biol.

[CR11] Hayden MS, Ghosh S (2012). NF-κB, the first quarter-century: remarkable progress and outstanding questions. Genes Dev.

[CR12] Heinz S, Benner C, Spann N, Bertolino E, Lin YC, Laslo P, Cheng JX, Murre C, Singh H, Glass CK (2010). Simple combinations of lineage-determining transcription factors prime cis-regulatory elements required for macrophage and B cell identities. Mol Cell.

[CR13] Hensen SMM, Heldens L, van Genesen ST, Pruijn GJM, Lubsen NH (2013). A delayed antioxidant response in heat-stressed cells expressing a non-DNA binding HSF1 mutant. Cell Stress Chaperones.

[CR14] Hess J, Angel P, Schorpp-Kistner M (2004). AP-1 subunits: quarrel and harmony among siblings. J Cell Sci.

[CR15] Hildebrandt B, Wust P, Ahlers O, Dieing A, Sreenivasa G, Kerner T, Felix R, Riess H (2002). The cellular and molecular basis of hyperthermia. Crit Rev Oncol Hematol.

[CR16] Inouye S, Fujimoto M, Nakamura T, Takaki E, Hayashida N, Hai T, Nakai A (2007). Heat shock transcription factor 1 opens chromatin structure of interleukin-6 promoter to facilitate binding of an activator or a repressor. J Biol Chem.

[CR17] Janus P, Pakuła-Cis M, Kalinowska-Herok M, Kashchak N, Szołtysek K, Pigłowski W, Widlak W, Kimmel M, Widlak P (2011). NF-κB signaling pathway is inhibited by heat shock independently of active transcription factor HSF1 and increased levels of inducible heat shock proteins. Genes Cells Devoted Mol Cell Mech.

[CR18] Jiang Q, Wang Y, Li T, Shi K, Li Z, Ma Y, Li F, Luo H, Yang Y, Xu C (2011). Heat shock protein 90-mediated inactivation of nuclear factor-κB switches autophagy to apoptosis through becn1 transcriptional inhibition in selenite-induced NB4 cells. Mol Biol Cell.

[CR19] Kampinga HH (2006). Cell biological effects of hyperthermia alone or combined with radiation or drugs: a short introduction to newcomers in the field. Int J Hyperth Off J Eur Soc Hyperthermic Oncol North Am Hyperth Group.

[CR20] Kampinga HH, Hageman J, Vos MJ, Kubota H, Tanguay RM, Bruford EA, Cheetham ME, Chen B, Hightower LE (2009). Guidelines for the nomenclature of the human heat shock proteins. Cell Stress Chaperones.

[CR21] Knowlton AA (2006). NFkappaB, heat shock proteins, HSF-1, and inflammation. Cardiovasc Res.

[CR22] Kretz-Remy C, Munsch B, Arrigo AP (2001). NFkappa B-dependent transcriptional activation during heat shock recovery. Thermolability of the NF-kappaB. Ikappa B complex. J Biol Chem.

[CR23] Kus-Liśkiewicz M, Polańska J, Korfanty J, Olbryt M, Vydra N, Toma A, Widłak W (2013). Impact of heat shock transcription factor 1 on global gene expression profiles in cells which induce either cytoprotective or pro-apoptotic response following hyperthermia. BMC Genom.

[CR24] Langmead B, Salzberg SL (2012). Fast gapped-read alignment with Bowtie 2. Nat Methods.

[CR25] Lee K-H, Hwang Y-H, Lee C-T, Kim YW, Han SK, Shim Y-S, Yoo C-G (2004). The heat-shock-induced suppression of the IkappaB/NF-kappaB cascade is due to inactivation of upstream regulators of IkappaBalpha through insolubilization. Exp Cell Res.

[CR26] Lee K-H, Lee C-T, Kim YW, Han SK, Shim Y-S, Yoo C-G (2005). Heat shock protein 70 negatively regulates the heat-shock-induced suppression of the IkappaB/NF-kappaB cascade by facilitating IkappaB kinase renaturation and blocking its further denaturation. Exp Cell Res.

[CR27] Matys V, Fricke E, Geffers R, Gössling E, Haubrock M, Hehl R, Hornischer K, Karas D, Kel AE, Kel-Margoulis OV, Kloos DU, Land S, Lewicki-Potapov B, Michael H, Munch R, Reuter I, Rotert S, Saxel H, Scheer M, Thiele S, Wingender E (2003). TRANSFAC: transcriptional regulation, from patterns to profiles. Nucleic Acids Res.

[CR28] Nivon M, Abou-Samra M, Richet E, Guyot B, Arrigo A-P, Kretz-Remy C (2012). NF-κB regulates protein quality control after heat stress through modulation of the BAG3-HspB8 complex. J Cell Sci.

[CR29] Page TJ, Sikder D, Yang L, Pluta L, Wolfinger RD, Kodadek T, Thomas RS (2006). Genome-wide analysis of human HSF1 signaling reveals a transcriptional program linked to cellular adaptation and survival. Mol BioSyst.

[CR30] Park K-J, Gaynor RB, Kwak YT (2003). Heat shock protein 27 association with the I kappa B kinase complex regulates tumor necrosis factor alpha-induced NF-kappa B activation. J Biol Chem.

[CR31] Perkins ND (2007). Integrating cell-signalling pathways with NF-kappaB and IKK function. Nat Rev Mol Cell Biol.

[CR32] Perkins ND (2012). The diverse and complex roles of NF-κB subunits in cancer. Nat Rev Cancer.

[CR33] Quinlan AR, Hall IM (2010). BEDTools: a flexible suite of utilities for comparing genomic features. Bioinforma Oxf Engl.

[CR34] Ran R, Lu AG, Zhang L, Tang Y, Zhu Y, Zhu HY, Xu HC, Feng YX, Han C, Zhou GP, Rigby AC (2004). Hsp70 promotes TNF-mediated apoptosis by binding IKK gamma and impairing NF-kappa B survival signaling. Genes Dev.

[CR35] Reimand J, Kull M, Peterson H, Hansen J, Vilo J (2007). g:Profiler–a web-based toolset for functional profiling of gene lists from large-scale experiments. Nucleic Acids Res.

[CR36] Rupik W, Jasik K, Bembenek J, Widłak W (2011). The expression patterns of heat shock genes and proteins and their role during vertebrate’s development. Comp Biochem Physiol A: Mol Integr Physiol.

[CR37] Salminen A, Paimela T, Suuronen T, Kaarniranta K (2008). Innate immunity meets with cellular stress at the IKK complex: regulation of the IKK complex by HSP70 and HSP90. Immunol Lett.

[CR38] Sheppard PW, Sun X, Khammash M, Giffard RG (2014). Overexpression of heat shock protein 72 attenuates NF-κB activation using a combination of regulatory mechanisms in microglia. PLoS Comput Biol.

[CR39] Singh IS, Viscardi RM, Kalvakolanu I, Calderwood S, Hasday JD (2000). Inhibition of tumor necrosis factor-alpha transcription in macrophages exposed to febrile range temperature. A possible role for heat shock factor-1 as a negative transcriptional regulator. J Biol Chem.

[CR40] Singh IS, He J-R, Calderwood S, Hasday JD (2002). A high affinity HSF-1 binding site in the 5′-untranslated region of the murine tumor necrosis factor-alpha gene is a transcriptional repressor. J Biol Chem.

[CR41] Singh IS, Gupta A, Nagarsekar A, Cooper Z, Manka C, Hester L, Benjamin IJ, He J-R, Hasday JD (2008). Heat shock co-activates interleukin-8 transcription. Am J Respir Cell Mol Biol.

[CR42] Smyth GK (2004). Linear models and empirical bayes methods for assessing differential expression in microarray experiments. Stat Appl Genet Mol Biol.

[CR43] Storey JD, Tibshirani R (2003). Statistical significance for genomewide studies. Proc Natl Acad Sci USA.

[CR44] Sun S-C (2012). The noncanonical NF-κB pathway. Immunol Rev.

[CR45] Takii R, Inouye S, Fujimoto M, Nakamura T, Shinkawa T, Prakasam R, Tan K, Hayashida N, Ichikawa H, Hai T, Nakai A (2010). Heat shock transcription factor 1 inhibits expression of IL-6 through activating transcription factor 3. J Immunol Baltim Md 1950.

[CR46] Tian B, Brasier AR (2003). Identification of a nuclear factor kappa B-dependent gene network. Recent Prog Horm Res.

[CR47] Tian B, Nowak DE, Brasier AR (2005). A TNF-induced gene expression program under oscillatory NF-kappaB control. BMC Genom.

[CR48] Trinklein ND, Murray JI, Hartman SJ, Botstein D, Myers RM (2004). The role of heat shock transcription factor 1 in the genome-wide regulation of the mammalian heat shock response. Mol Biol Cell.

[CR49] Vallabhapurapu S, Karin M (2009). Regulation and function of NF-kappaB transcription factors in the immune system. Annu Rev Immunol.

[CR50] Vertegaal AC, Kuiperij HB, Yamaoka S, Courtois G, van der Eb AJ, Zantema A (2000). Protein kinase C-alpha is an upstream activator of the IkappaB kinase complex in the TPA signal transduction pathway to NF-kappaB in U2OS cells. Cell Signal.

[CR51] Voellmy R (2004). On mechanisms that control heat shock transcription factor activity in metazoan cells. Cell Stress Chaperones.

[CR52] Vydra N, Toma A, Widlak W (2014). Pleiotropic role of HSF1 in neoplastic transformation. Curr Cancer Drug Targets.

[CR53] Weiss YG, Bromberg Z, Raj N, Raphael J, Goloubinoff P, Ben-Neriah Y, Deutschman CS (2007). Enhanced heat shock protein 70 expression alters proteasomal degradation of IkappaB kinase in experimental acute respiratory distress syndrome. Crit Care Med.

[CR54] Wilson CL, Miller CJ (2005). Simpleaffy: a BioConductor package for Affymetrix quality control and data analysis. Bioinforma Oxf Engl.

[CR55] Wong HR, Ryan M, Wispé JR (1997). Stress response decreases NF-kappaB nuclear translocation and increases I-kappaBalpha expression in A549 cells. J Clin Invest.

[CR56] Wong HR, Ryan M, Wispé JR (1997). The heat shock response inhibits inducible nitric oxide synthase gene expression by blocking I kappa-B degradation and NF-kappa B nuclear translocation. Biochem Biophys Res Commun.

[CR57] Wu Z, Irizarry R, Gentleman R, Murillo FM, Spencer F (2004). A model based background adjustment for oligonucleotide expression arrays. J Amer Stat Assoc.

[CR58] Zheng Z, Kim JY, Ma H, Lee JE, Yenari MA (2008). Anti-inflammatory effects of the 70 kDa heat shock protein in experimental stroke. J Cereb Blood Flow Metab Off J Int Soc Cereb Blood Flow Metab.

