# Supplementary figures and images for: Cross talk between cytokine and hyperthermia-induced pathways: identification of different subsets of NF-κB-dependent genes regulated by TNFα and heat shock
Source: Mol Genet Genomics. 2015 May 6;290:1979–90. doi: 10.1007/s00438-015-1055-1 (PMC4768219; doi:10.1007/s00438-015-1055-1)

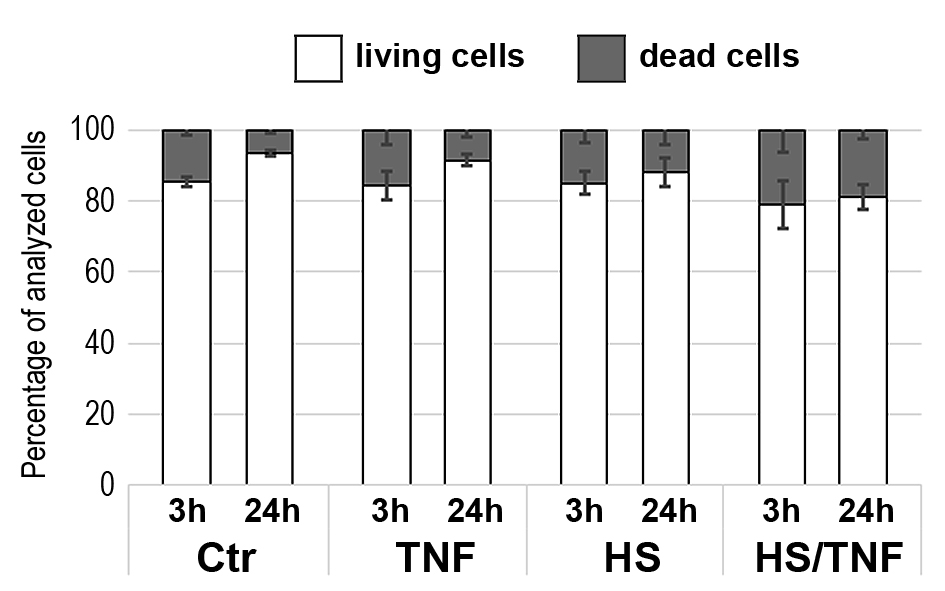

Supplement: Supplementary file 1 — Supplementary material 1 Toxicity of heat shock and/or stimulation with TNFα cytokine to U-2 OS cells. Cells were collected 3 and 24 h after the end of HS (1 h at 43 °C), or at corresponding time points in untreated controls or cells stimulated with cytokine. Relative numbers of living and dead cells were assessed by flow cytometry after staining with propidium iodide; shown are the mean values ± SD based on three experiments (JPEG 154 kb) [file 438_2015_1055_MOESM1_ESM.jpg]

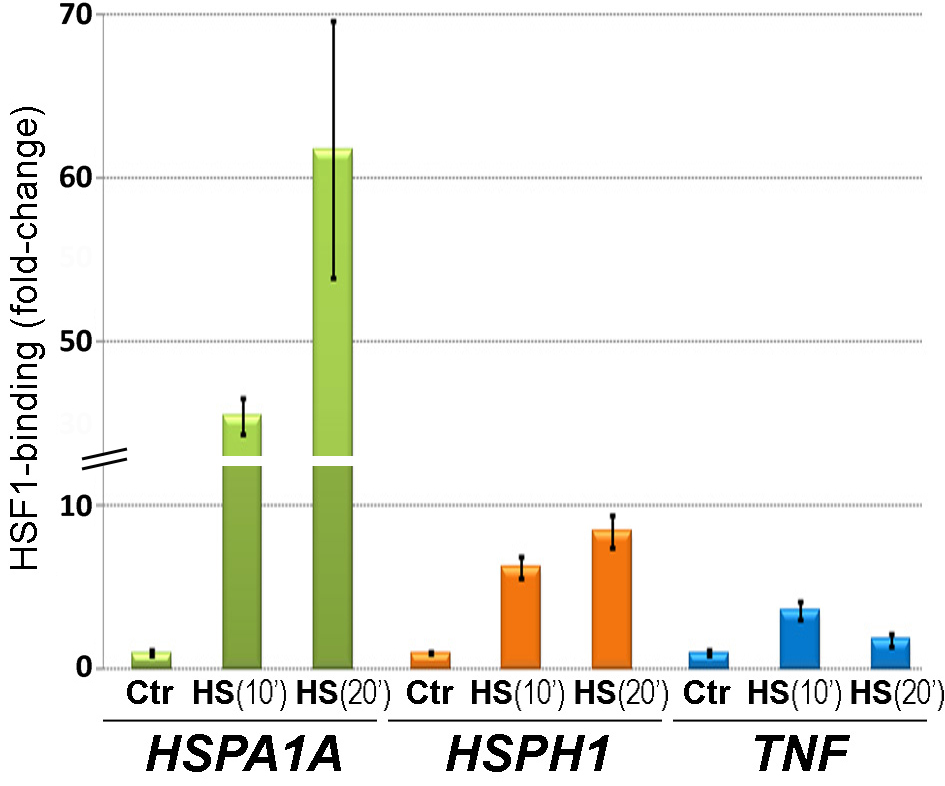

Supplement: Supplementary file 2 — Supplementary material 2 Binding of HSF1 in promoters of HSPA1A, HSPH1, and TNF genes. Relative binding of HSF1 (assessed by ChIP-QPCR) is shown as a fold-change against untreated control (Ctr); cells were analyzed after 10 and 20 min of heat shock—HS(10′) and HS(20′), respectively. Shown are the mean values ± SD based on three experiments (JPEG 168 kb) [file 438_2015_1055_MOESM2_ESM.jpg]

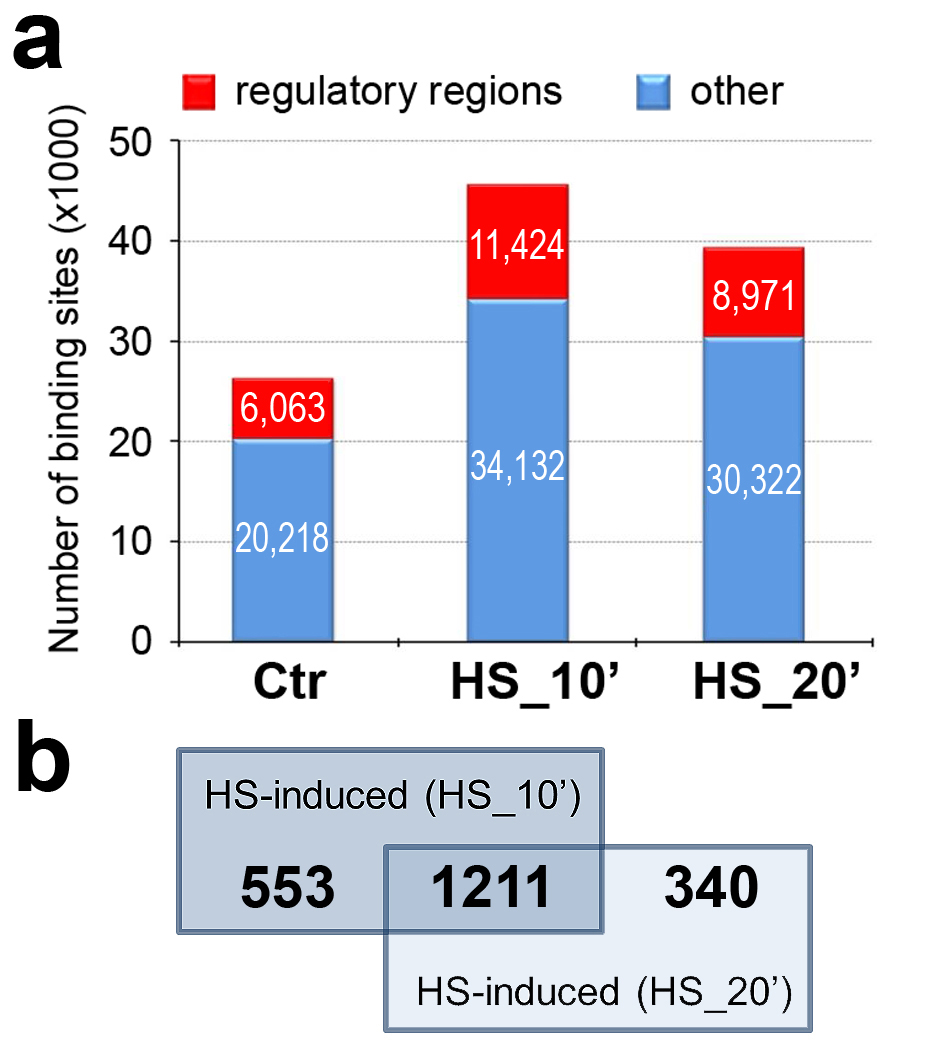

Supplement: Supplementary file 3 — Supplementary material 3 Numbers of functional HSF1 binding sites in chromatin of U-2 OS cells. a Total number of HSF1 binding sites in untreated control cells (Ctr) and cells exposed to heat shock for 10 or 20 min––HS_10′ and HS_20′, respectively; depicted are binding sites present in potential regulatory regions (from −2500 to +500 bp from TSS) and other genomic sites. b Heat shock-induced HSF1 binding in regulatory regions after either 10 or 20 min of heat shock; depicted are numbers of genes with binding sites where HSF1 binding was significantly increased compared to untreated control (FDR <0.05) (JPEG 240 kb) [file 438_2015_1055_MOESM3_ESM.jpg]

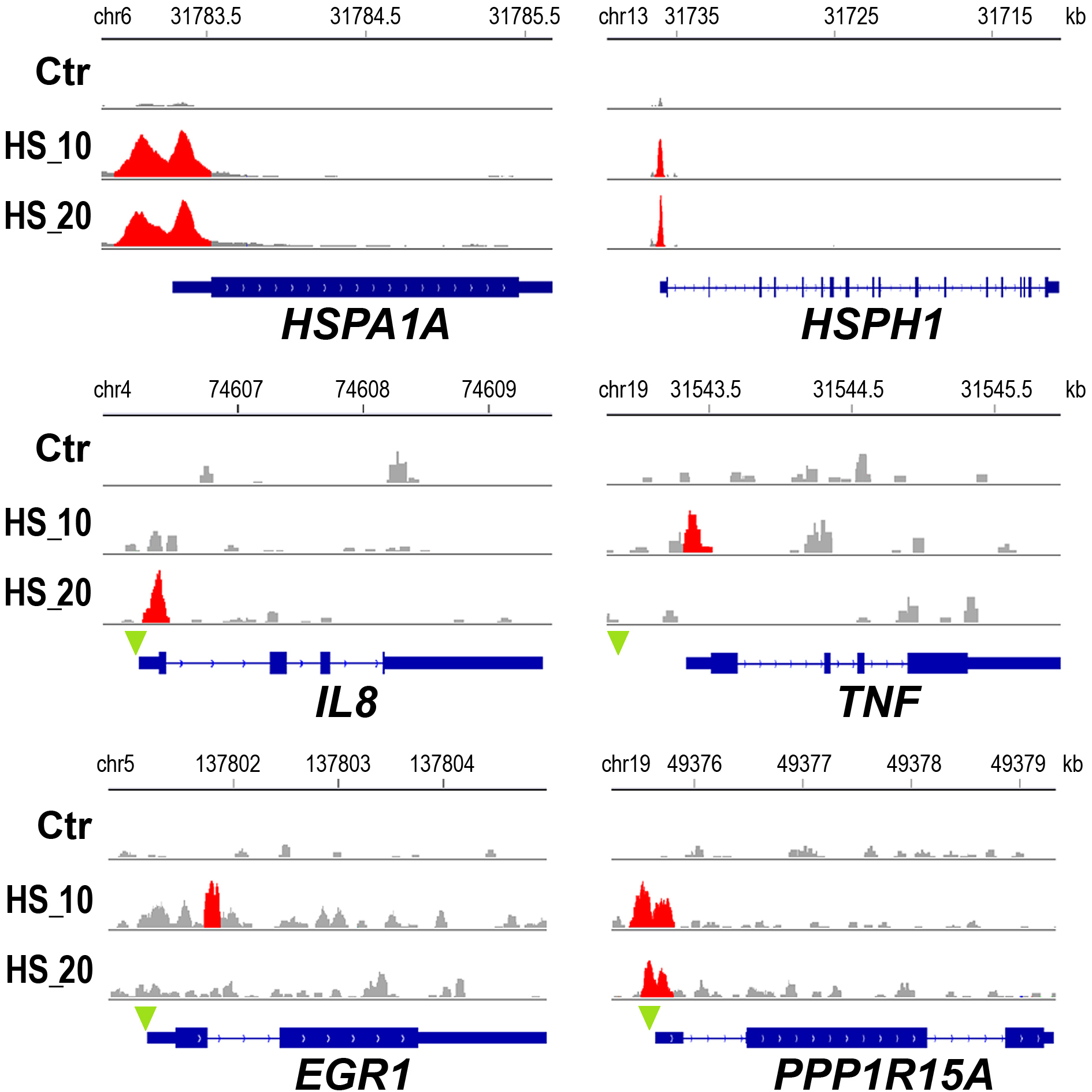

Supplement: Supplementary file 4 — Supplementary material 4 Heat shock-induced HSF1 binding sites in regulatory regions of HS-responsive genes HSPA1A and HSPH1, and TNF-responsive genes IL8, TNF, EGR1 and PPP1R15A. Shown are HSF1 peaks detected by ChIP-Seq in control and heat-shocked cells (HS_10 and HS_20), exons (represented by thick blue bars) and NF-κB binding sites (represented by green arrowheads); statistically significant HS-induced HSF1 binding sites are marked in red. (JPEG 464 kb) [file 438_2015_1055_MOESM4_ESM.jpg]

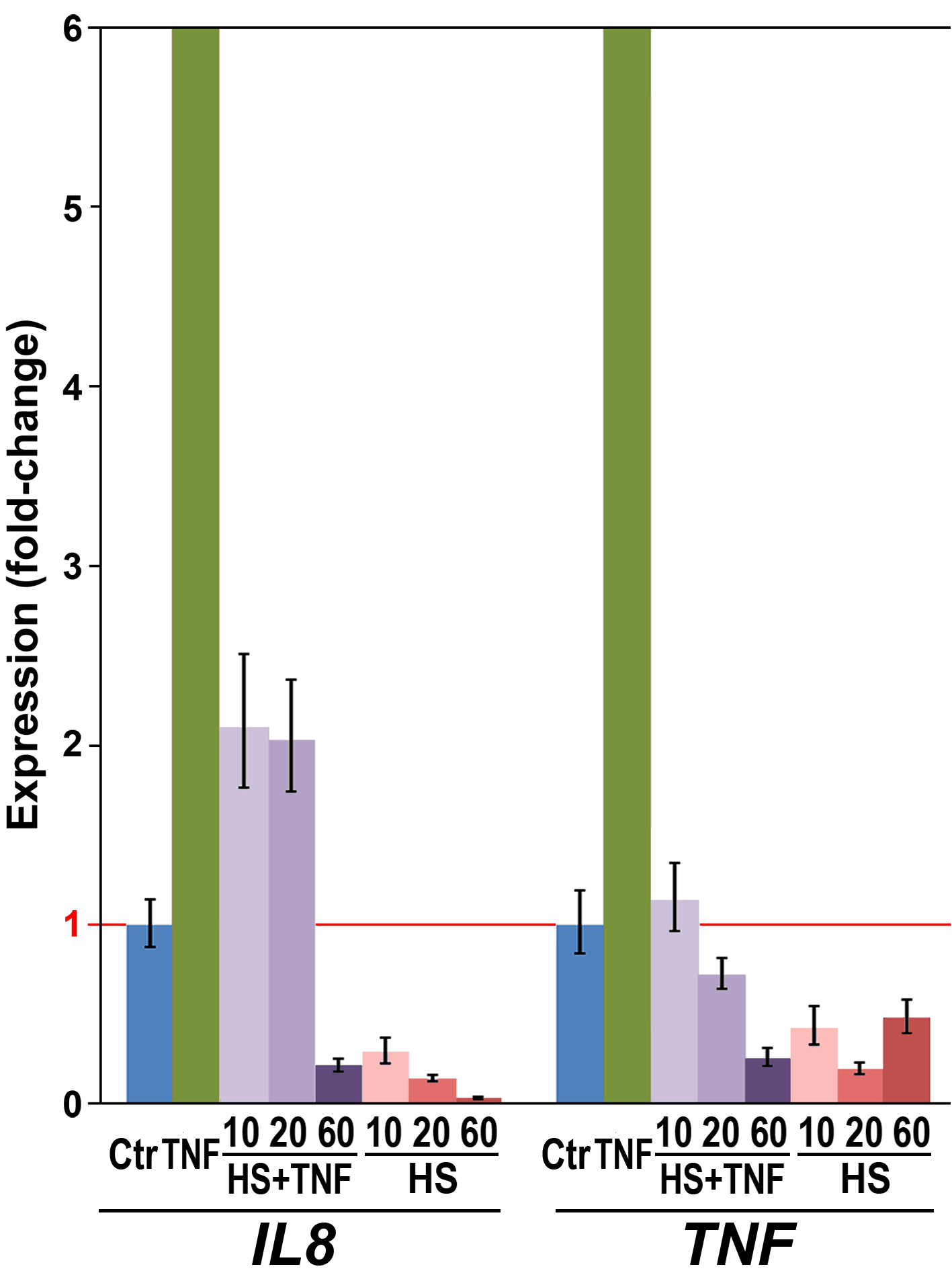

Supplement: Supplementary file 5 — Supplementary material 5 The influence of heat shock extension on expression TNF-upregulated genes. Cells were stimulated with TNFα alone (TNF), heat-shocked at 43 °C for 10, 20 or 60 min, and then stimulated with TNFα (HS + TNF), or treated with HS alone for 10, 20 or 60 min. Expression of IL8 and TNF genes was assessed by QRT-PCR and presented as a fold-change against untreated controls (Ctr); shown are the mean values ± SD (JPEG 242 kb) [file 438_2015_1055_MOESM5_ESM.jpg]
