# Supplementary material for: Cross talk between cytokine and hyperthermia-induced pathways: identification of different subsets of NF-κB-dependent genes regulated by TNFα and heat shock
Source: Mol Genet Genomics. 2015 May 6;290:1979–90. doi: 10.1007/s00438-015-1055-1 (PMC4768219; doi:10.1007/s00438-015-1055-1)
Supplement: Supplementary file 6 — Supplementary material 6 (DOC 47 Kb) [file 438_2015_1055_MOESM6_ESM.doc]

I Primer pairs for promoter-specific quantitative real-time PCR (ChIP-PCR)

| **Binding sites** | **Gene** | **Genomic sequence** | **Product size** | **Sense primer** | **5’ position from TSS** | **Antisense primer** | **5’ position from TSS** |
| --- | --- | --- | --- | --- | --- | --- | --- |
| **HSE** | *TNF* | NC_000006.11 | 247 bp | attctttccccgccctcctc | -71 | tgctcatggtgtcctttcca | +176 |
|  | *HSPA1A* | NC_000001.10 | 128 bp | cggcactctggcctctgatt | -141 | gacccgccttttcccttctg | -14 |
|  | *HSPH1* | NC_000013.10 | 151 bp | tgcccattgggtagaatctttc | -82 | gaggtcccacttcctcagcctta | +69 |
| **κB** | *EGR1* | NC_000005.9 | 63 bp | gccgttccagacccttcaaat | -56 | tctgcgcgctgggatctct | +6 |
|  | *FOSB* | NC_000019.9 | 93 bp | cctctgacgtcattgctaggat | -328 | cttcccggagttcctgtgac | 236 |
|  | *PPP1R15A* | NC_000019.9 | 86 bp | gcatttgattgacagttcgtttg | -181 | cgcggagattccagctttt | 96 |
|  | *CD83* | NC_000006.11 | 115 bp | cggcctaagcgggactagga | +14 | cgccggctgcccttttat | +128 |
|  | *IL8* | NC_000004.12 | 183 bp | gggccatcagttgcaaatc | -121 | ttccttccggtggtttcttc | +61 |
|  | *TNF* | NC_000006.11 | 114 bp | ccttggaagccaagactgaaacc | -711 | agccccggggagtgaaat | -598 |
|  |  |  |  |  |  |  |  |
| **none** | *Neg. CTR* | NT_009759.16 | 174 bp | atggttgccactggggatct |  | tgccaaagcctaggggaaga |  |

II Primer pairs for gene-specific quantitative real-time PCR (qRT-PCR)

| **Gene** | **mRNA sequence** | **Product size** | **Sense primer** | **5’ position from ATG** | **Antisense primer** | **5’ position from ATG** |
| --- | --- | --- | --- | --- | --- | --- |
| ***EGR1*** | NM_001964.2 | 181 bp | agcaacagcagcagcagca | 178 | tggtctccaccagcaccttc | 358 |
| ***FOSB*** | NM_001114171.1 | 156 bp | aaccagctactccacaccaggc | 303 | tgatctgtctccgtctcctctcg | 458 |
| ***PPP1R15A*** | NM_014330.3 | 143 bp | aagctgctgaggactgggga | 1481 | aggggtttctgggcgcttga | 1623 |
| ***CD83*** | NM_001040280.1 | 161 bp | gccctgcacagcgtaaagaaga | 386 | agctcgttccatgccagctt | 546 |
| ***IL8*** | NM_000584.2 | 145 bp | gcagctctgtgtgaaggtgcag | 49 | ctgtgttggcgcagtgtggt | 193 |
| ***TNF*** | NM_000594.3 | 167 bp | aggccttcctctctccagatg | 1067 | ggataccccggtctcccaaa | 1233 |
